# Comparative Transcriptomic Profiling of Two Tomato Lines with Different Ascorbate Content in the Fruit

**DOI:** 10.1007/s10528-012-9531-3

**Published:** 2012-08-22

**Authors:** Antonio Di Matteo, Adriana Sacco, Rosalba De Stefano, Luigi Frusciante, Amalia Barone

**Affiliations:** Department of Soil, Plant, Environmental and Animal Sciences, University of Naples “Federico II”, Via Università 100, 80055 Portici, Italy

**Keywords:** Antioxidants, Berry quality, Carbohydrate metabolism, *Solanum* spp., Reactive oxygen species

## Abstract

In recent years, interest in tomato breeding for enhanced antioxidant content has increased as medical research has pointed to human health benefits from antioxidant dietary intake. Ascorbate is one of the major antioxidants present in tomato, and little is known about mechanisms governing ascorbate pool size in this fruit. In order to provide further insights into genetic mechanisms controlling ascorbate biosynthesis and accumulation in tomato, we investigated the fruit transcriptome profile of the *Solanum pennellii* introgression line 10-1 that exhibits a lower fruit ascorbate level than its cultivated parental genotype. Our results showed that this reduced ascorbate level is associated with an increased antioxidant demand arising from an accelerated oxidative metabolism mainly involving mitochondria, peroxisomes, and cytoplasm. Candidate genes for controlling ascorbate level in tomato fruit were identified, highlighting the role of glycolysis, glyoxylate metabolism, and purine breakdown in modulating the ascorbate pool size.

**Electronic supplementary material:**

The online version of this article (doi:10.1007/s10528-012-9531-3) contains supplementary material, which is available to authorized users.

## Introduction

In recent years, the compositional quality of crops has received increasing interest, particularly given the results of recent studies highlighting human health benefits brought about by antioxidants (Chu et al. 2002; Naidu 2003; Stanner et al. 2004). The tomato (*Solanum lycopersicum* Mill.) represents a major contribution to dietary nutrition worldwide, and its beneficial effects are generally attributed to its antioxidant content. Its antioxidant compounds include ascorbate (also known as vitamin C), which is an essential nutrient for humans, primates, and a number of other animals that have lost the ability to synthesize it because of inactivation of the last enzyme in the pathway. In plants, ascorbate accumulates at intracellular concentrations of 2–25 mM (Davey et al. 2000) and acts as an antioxidant, as a cofactor for various enzymes, and as a contributor to the regulation of cell division and expansion (Smirnoff and Wheeler 2000). As a signaling agent, ascorbate participates in the interaction with the environment, pathogens and oxidizing agents, and water loss (Pastori et al. 2003; Sanmartin et al. 2003; Fotopoulos et al. 2006). It is essential for plant growth, participates in stress resistance, and seems to control flowering time and senescence (Davey et al. 2000).

Plants synthesize ascorbate through alternative biosynthetic pathways (Valpuesta and Botella 2004). In particular, the Wheeler–Smirnoff pathway operates through l-galactose as a key intermediate. In addition, l-gulose and myo-inositol have been proposed as intermediates in ascorbate biosynthesis, and an l-galactonic acid intermediate has also been reported. The simultaneous operation of these pathways has been demonstrated only in *Arabidopsis*, but their physiological relevance still has to be demonstrated in vivo. Independent of the biosynthetic pathways, the reduced ascorbate is oxidized into an unstable radical, monodehydroascorbate (MDA), which dissociates into ascorbate and dehydroascorbate (DHA). DHA undergoes irreversible hydrolysis to 2,3-diketogulonic acid or is recycled to ascorbate by DHA reductase, which uses glutathione as the reductant, whereas MDA reductase can recycle MDA to ascorbate. This pathway, also called the Foyer–Halliwell–Asada cycle, is an efficient way to control hydrogen peroxide and recycle ascorbate using glutathione as the electron donor (Halliwell and Gutteridge 2000). Overall, the regulation of ascorbate levels in cells is tightly controlled by the level of synthesis, recycling, degradation, and transport of this molecule within the cell or between organs (Hancock and Viola 2005).

Very little is known about the relative contribution of alternative pathways for ascorbate biosynthesis operating in ripening tomato fruit and mechanisms governing the ascorbate pool size in this fruit (Zou et al. 2006; Ioannidi et al. 2009; Di Matteo et al. 2010; Haroldsen et al. 2011). Therefore, understanding such mechanisms will provide an opportunity to breed nutritional quality and enhance postharvest quality. A trait exhibiting quantitative variation, ascorbate content is controlled by several genes and is more or less influenced by the environment (Stevens et al. 2007), and it thus lends itself to quantitative trait loci (QTL) analysis. One approach to identify QTLs controlling ascorbate accumulation in tomato is the use of introgression lines (ILs). These are homozygous lines with single chromosome segment substitutions from one wild relative (Eshed and Zamir 1995). In addition, the combined use of ILs and transcriptomic profiling (Barone et al. 2009) could be effective in rapidly identifying transcriptional networks and candidate genes involved in fruit antioxidant accumulation.

In order to provide additional insights into genetic mechanisms controlling fruit quality in tomato fruit, in a previous work (Di Matteo et al. 2010) we investigated the fruit transcriptome of a *Solanum pennellii* introgression line (IL12-4) that exhibited a higher ascorbate content than the control variety, M82. This work highlighted the link between genes associated with cell wall catabolism, ethylene, and genes involved in ascorbate pathways. Thus, to explore further mechanisms controlling fruit quality traits in tomato, in the present work we investigated the fruit transcriptome of an additional introgression line (IL10-1) that produces a lower level of ascorbate than the parental cultivated variety M82. In particular, genes and molecular networks mapping to glyoxylate pathways have been involved for the first time in controlling the ascorbate level in tomato fruit. This is discussed according to a model that explains the control of ascorbate level by regulating the steady-state level of specific mRNAs. Indeed, candidate mRNAs in controlling ascorbate level in IL10-1 reported here are expected to drive new strategies of precision breeding aimed at engineering the tomato for quality fruit.

## Materials and Methods

### Plant Material

The tomato introgression line IL10-1 and its parental genotypes *S. lycopersicum* cv. M82 and *S. pennellii* were cultivated over three consecutive years (2006–2008) in a greenhouse at the Department of Soil, Plant, Environmental, and Animal Production Sciences at the University of Naples (Portici, Italy), as previously described (Di Matteo et al. 2010). IL10-1 (accession LA4102) is a green shoulder red-fruited tomato containing a 43 cM homozygous introgression from *S. pennellii* (acc. LA0716) in an *S. lycopersicum* cv. M82 background (acc. LA3475) (Eshed and Zamir 1995). LA0716 is a homozygous, self-fertile indeterminate accession from Atico, Peru, with green fruits. M82 is a determinate, red-fruited tomato used for processing. All seeds were provided by the C. M. Rick Tomato Genetics Resource Center at the University of California (Davis).

Fruits were collected from IL10-1 and its cultivated parent when 75 % were full sized and red-ripe, softening had increased, and the inside of the columella was completely red. For both lines, three samples were collected within each year. Samples were generated by pooling ripe fruit from the same plant and discarding the seeds, jelly parenchyma, columella, and placenta tissues. Frozen samples under liquid nitrogen were stored at −80 °C prior to homogenization in a Waring blender and processing for the extraction of total RNA and ascorbate.

### Phenotypic Evaluation

Ascorbate levels in the pericarp of red-ripe fruit were measured using the procedure described by Di Matteo et al. (2010). Statistical analysis was performed using SPSS 15.0 for Windows (evaluation version release 15.0.0). The significance of genotype with respect to ascorbate level in fruit over three consecutive greenhouse trials was determined by comparing mean levels in IL10-1 and M82 samples using a univariate ANOVA. Because ascorbate revealed a significant interaction between genotype and year (*P* < 0.05), an independent-sample Student’s *t*-test was used to compare IL10-1 to the M82 reference within each trial.

### Transcriptomic Analysis

A microarray experiment was designed and conducted according to the MIAME guidelines (www.mged.org/miame) on a 90K TomatArray 1.0 microarray synthesized using the CombiMatrix platform at the Plant Functional Genomics Center of the University of Verona (http://ddlab.sci.univr.it/FunctionalGenomics/), as previously described by Di Matteo et al. (2010).

Total RNA used for downstream microarray hybridization and qPCR validation was extracted from frozen, homogenized, and powdered fruit tomato samples of genotypes IL10-1 and M82 using the CTAB (hexadecyltrimethylammonium bromide) method described by Griffiths et al. (1999). Within each line, three samples per trial (2007 and 2008) were assembled and each sample was obtained by pooling 3-5 red-ripe fruit from a single plant. Antisense RNA (aRNA) was obtained using the SuperScript Indirect RNA Amplification System Kit (Invitrogen), and labeling was performed by incorporating Alexa Fluor 647 Reactive Dye. Prehybridization, RNA fragmentation, hybridization with 3 μg labeled and fragmented aRNA, and posthybridization washes were performed according to CombiMatrix protocols (http://www.combimatrix.com/docs/PTL020_00_90K_Hyb_Imaging.pdf).

Imaging of the microarray slides was performed using a Perkin Elmer ScanArray 4000XL and the accompanying acquisition software (ScanArray Express Microarray Analysis System version 4.0). The resulting TIFF images were processed to extract raw data using the CombiMatrix Microarray Imager software version 5.8.0. Signal probe medians and standard deviations were imported into the SPSS software, and normalization was achieved by correcting each probe mean based on the ratio between the median of the array and the average median of arrays. Following data normalization and quality control, all values were log transformed (base 2). Finally, probe signals with a variability coefficient higher than 0.5, as well as spikes and factory probes, were filtered out. Also, probes with signal intensities in the uppermost and lowermost 10 % of values were deleted. The microarray data were deposited in the Gene Expression Omnibus under the series accession GSE26962. Differential signals in the IL10-1 versus M82 fruit transcriptomes were identified using the two-factor ANOVA module in the TIGR MultiExperiment Viewer version 4.5 (MeV, part of the TM4 software suite at http://www.tm4.org/mev/). Because small changes in gene expression might underlie differences in ascorbate accumulation, differentially expressed transcripts were not filtered using a fold-change threshold, and differences were considered irrespective of the intensity of the change. Hierarchical clustering of differentially expressed signals was achieved using the Pearson correlation as a metric to investigate gene expression coregulation. In addition, a relevance network analysis was carried out on microarray normalized data from all 233 probes that previously showed statistically significant variation in their expression, as a result of running the two-factor ANOVA module. The network was generated using the Pearson correlation as the metric and retaining only probes correlated with an *R* ^2^ range of 0.92–1.0.

Blast2GO (http://blast2go.bioinfo.cipf.es/) was used to provide automatic high throughput annotation, gene ontology mapping, and categorization of tentative consensus (TC) transcripts showing differential transcription signals. Manually curated annotation was performed for those sequences that were not automatically annotated through similarity matching in the NCBI’s nonredundant database. In particular, a number of sequences were processed manually using the similarity search tools BlastX (http://blast.ncbi.nlm.nih.gov/Blast.cgi) and/or SGN Blast (http://sgn.cornell.edu/tools/blast/). In each case, an expectation threshold of 10^−10^ was used. Sequences were also mapped to chromosomes on the tomato genome sequence release 2.40 by performing a multi-Blast search in the Sol Genomics Network Database (http://sgn.cornell.edu/tools/blast).

The expression profiles of a group of TC transcripts considered to be key control points for ascorbate accumulation were validated by real-time quantitative RT-PCR in a 7900HT Fast Real-Time PCR System (Applied Biosystems). Moreover, to assay the reproducibility of the microarray experiment, we performed RT-qPCR on 86 transcripts; 69 of them confirmed the expression pattern shown by microarray analysis. Primer pairs (Table 1) were validated using a standard curve over a dilution range of 1–10^−3^ (*R* ^2^ > 0.98; slope close to −3.32). Amplification was performed in 12.5 μl reaction volumes using a Power SYBR Green PCR Master Mix (Applied Biosystems). Relative quantification was achieved by the ΔΔC_T_ method (Livak and Schmittgen 2001). The assembly of reactions in a 96-well plate format was automated with a Tecan FreedomEvo 150 liquid handler.

**Table 1:** Primer pairs used for qPCR validation of genes involved in ascorbate accumulation

TIGR ID	Forward primer	Reverse primer
TC178207	5′-ggatgcaagtggatatgctg-3′	5′-gaaatgaggatggtgttctgg-3′
TC177287	5′-tcccatgctgaggcaacttc-3′	5′-gggcaattccatctccaagag-3′
TC182471	5′-gccatccattggcattcct-3′	5′-tgaaccattagcccagtggg-3′
TC180820	5′-ccaagccattgaattagcatt-3′	5′-caggcctcggtagcaatatg-3′
TC190330	5′-tggcgaaagaggaatctgtt-3′	5′-tccagttcttcaaacccacag-3′
TC190409	5′-cgtttgccaagtaaccaaca-3′	5′-catgactcgtaatggtcgtatca-3′
TC172563	5′-cgtatccccgtgttcctgg-3′	5′-gcccaataaacacgcctgat-3′

## Results

### Phenotypic Characterization

**Fig. 1:**
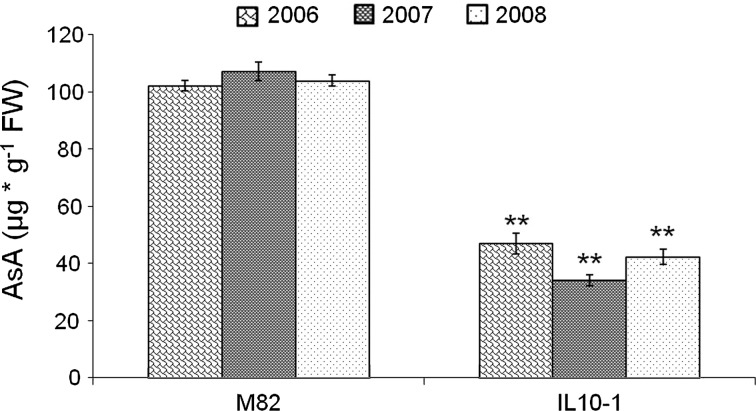
Ascorbate concentration in red-ripe fruit from the tomato lines M82 and IL10-1. Concentrations are expressed as μg g^−1^ fresh weight (FW); mean values ± SE are reported for three years (2006, 2007, 2008); **statistically significant difference at *P* < 0.001, Student’s *t*-test

Compared with the parental line M82, IL10-1 showed a significantly reduced level of fruit ascorbate content over three consecutive trials (Fig. 1). The average ascorbate concentration in red-ripe M82 fruit was 122 μg g^−1^ fresh weight, whereas in IL10-1 it was 62 μg g^−1^ fresh weight. This difference was statistically significant (univariate ANOVA procedure; *F*_1,31_ = 56.56; *P* < 0.001). A significant difference among years was also observed (*F*_2,31_ = 3.33; *P* < 0.05), but the interaction genotype × year over the three consecutive trials was not significant (*F*_2,31_ = 0.441; *P* > 0.05). Within each trial, the ascorbate level in the IL10-1 fruit was significantly lower than in M82 (Student’s *t*-test, *P* < 0.001). Indeed, the ascorbate content on average was 58 % lower in IL10-1 fruit than in M82 in 2006, 53 % lower in 2007, and 40 % lower in 2008. Therefore, the introgression from the *S. pennellii* genome into IL10-1 contributes to lower fruit ascorbate content at the red-ripe stage, providing evidence that a QTL in this region negatively affects ascorbate concentration in the fruit.

### Comparative Microarray Analysis

Transcriptomic analysis revealed 233 mRNA sequences that were differentially expressed between IL10-1 and M82 (Supplementary Table 1). These sequences accounted for 1.15 % of those represented on the TomatArray 1.0 chip; 84 (36 %) of them were upregulated and 149 (63.9 %) were downregulated in IL10-1. Of the differentially expressed sequences, 18 % significantly matched (*e* value < 1 × 10^−10^) with sequences generically annotated as protein in the NCBI’s nonredundant database, whereas 6.9 % showed no matches and were thus reported as nonannotated. They were distributed in GO categories according to biological process, molecular function, and cellular component vocabularies (Supplementary Figs. 1–3).

Even though the TomatArray 1.0 chip contains an exhaustive coverage of expressed sequences related to ascorbate alternative biosynthetic pathways, oxidation, and recycling, including those reported by Zou et al. (2006) and Ioannidi et al. (2009), our experiment did not identify differences in the expression of any of these sequences, as was also confirmed by qPCR validations carried out on a set of genes belonging to these pathways (data not shown). This suggests that reduced ascorbate level in IL10-1 fruit was not affected by transcriptional mechanisms operating within ascorbate biosynthetic pathways.

To gain additional insights on transcriptional mechanisms controlling ascorbate accumulation, we used hierarchical clustering and relevance networks to investigate the correlation among differentially expressed transcripts. In the cluster analysis, cluster 2 contains upregulated sequences in IL10-1 fruit linking genes involved in carbohydrate (glycolysis and the citric acid cycle) and amino acid catabolism with ethylene and defense responses, whereas cluster 5 mainly includes sequences related to peroxisome metabolism and biogenesis, such as a glycolate oxidase and a peroxisomal biogenesis factor (Supplementary Fig. 4). The relevance networks visually link 22 TC transcripts with strongly correlated transcriptional patterns (Supplementary Fig. 5). In particular, 17 of the sequences mapped to the introgression 10-1, 3 sequences to chromosome 10 outside the introgression 10-1 (TC179505, TC189885, and TC177168), and one each to chromosomes 1 (TC180796) and 12 (TC174331).

**Fig. 2:**
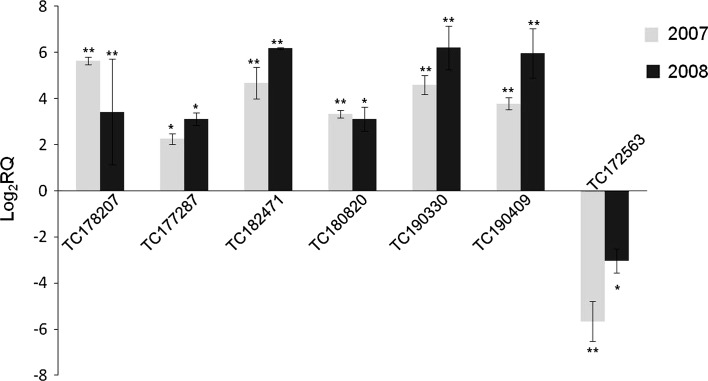
Validation of differential expression of seven TC transcripts by qRT-PCR. The relative quantification (RQ) of transcripts between tomato lines IL10-1 and M82 was evaluated in years 2007 (*gray bar*) and 2008 (*black bar*). Mean values ± SE are shown. *Asterisks* indicate statistically significant differences: *0.01 < *P* < 0.05; **P < 0.01; Student’s *t*-test

Altogether, these transcripts could be relevant key elements in reducing ascorbate content in IL10-1 red-ripe fruit. Indeed, based on functional annotation, gene ontology classification, clustering, and networking, a subset of the 233 differentially expressed TC transcripts (Table 2) was selected to develop a model that could explain the lower ascorbate content in IL10-1. Within this selected group, four transcripts fell in glycolysis, five in fatty acid biosynthesis, five in the tricarboxylic acid cycle, five in glyoxylate metabolism, and six in the antioxidant system. Most transcripts relevant to explaining the IL10-1 fruit phenotype were also validated in their expression pattern by real-time RT-qPCR (Fig. 2). Besides those, 89 more transcripts were analyzed, and 69 of them confirmed their pattern of expression according to microarray analysis, thus revealing 80 % concordance between data obtained from microarray and RT-qPCR analyses.

**Table 2:** Differential expression of transcripts involved in the genetic control of ascorbate content in fruit of tomato lines IL10-1 and M82

TIGR ID	IL10-1 versus M82 expression^a^	Chromosome location^b^	Cluster^c^	Annotation
Glycolysis				
TC172484	+	10^b^	2	Pyruvate decarboxylase
TC178207	+	10^b^	2	Pyruvate decarboxylase
TC186449	+	6	2	Phosphoglycerate mutase
TC187142	+	1	2	Acetyl-CoA synthetase
Fatty acid biosynthesis				
TC171885	−	9	5	Fatty acid elongase-like protein
TC172218	+	10^b^	2	Non-specific lipid transfer protein
TC177287	+	1	2	Wax synthase
TC180054	+	6	2	Stearoyl-acp desaturase
TC182471	+	1	2	Wax synthase
Tricarboxylic acid cycle				
TC170372	+	10^b^	2	NAD^+^-dependent isocitrate dehydrogenase subunit 1
TC180820	+	10^b^	2	Tyrosine aminotransferase
TC190330	+	10^b^	2	Tyrosine aminotransferase
TC190409	+	7	2	Aspartate aminotransferase
TC190777	+	1	2	Glutamate decarboxylase
Glyoxylate metabolism and peroxisome-related processes				
TC172563	−	10^b^	5	Glycolate oxidase
TC172641	+	10^b^	2	Endoribonuclease l-psp family protein
TC178654	+	2	2	Allantoate amidinohydrolase
TC187192	−	1	3	ATP-citrate lyase a-3
TC188826	−	10^b^	6	Gluconokinase
Antioxidant system				
TC175968	−	1	1	Glutathione *S*-transferase
TC177168	−	10	5	Thioredoxin m
TC179606	−	10^b^	3	NADH dehydrogenase
TC182153	−	1	3	Glutathione *S*-transferase
TC185135	−	10^b^	6	Thioredoxin m
TC189778	−	1	1	Glutathione *S*-transferase

^a^According to microarray results; + upregulated, − downregulated

^b^Physically mapping on introgression 10-1 of the tomato genome; release 2.40

^c^From hierarchical clustering in Supplementary Fig. 4

**Fig. 3:**
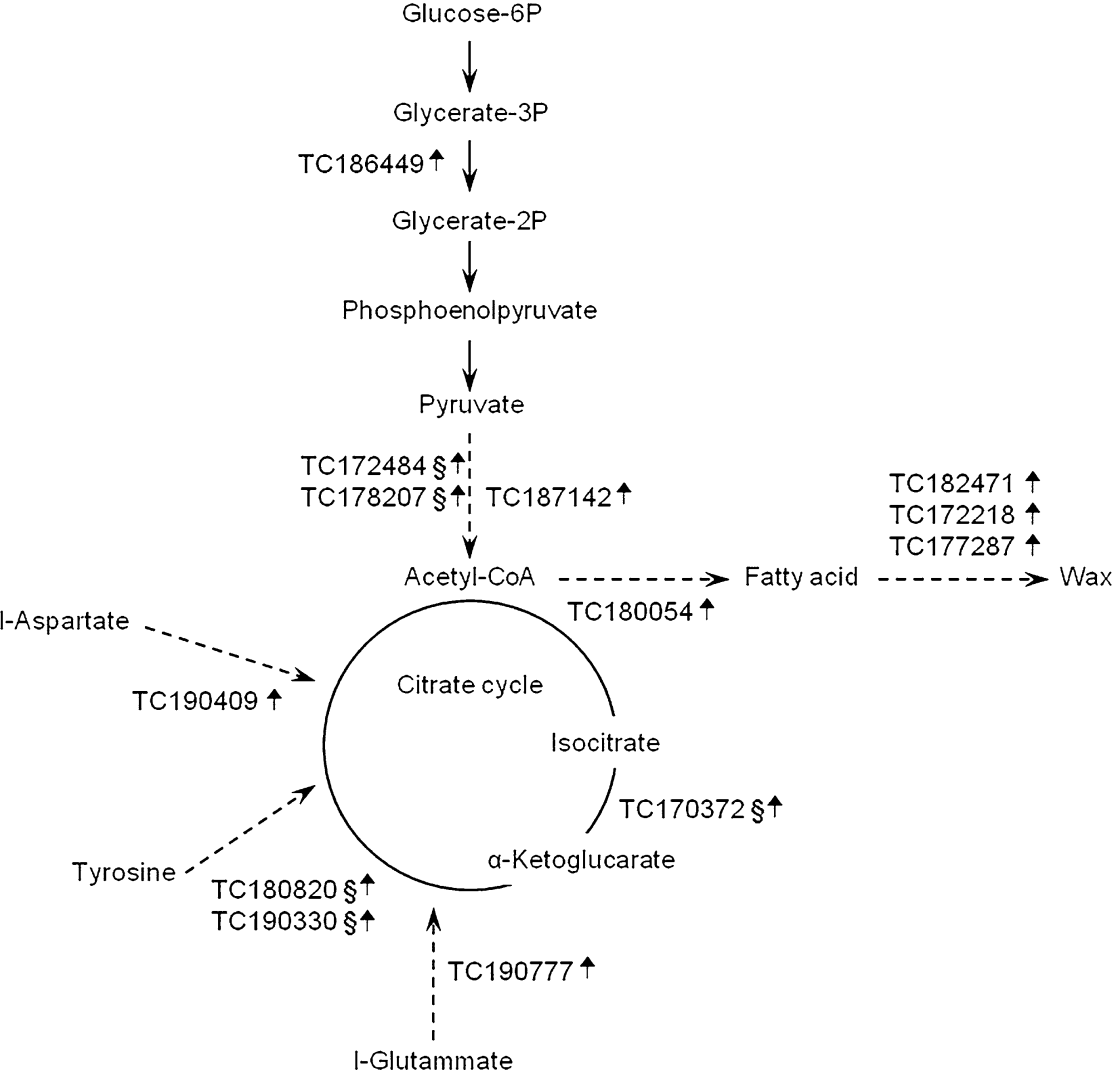
Network of genes mapping to glycolysis, fatty acid, and citrate cycle metabolism, illustrating changes in their expression between IL10-1 and M82. §, Transcript mapping on the introgression 10-1

As for transcripts likely involved in glycolysis (Fig. 3), the microarray experiment revealed the upregulation of a phosphoglycerate mutase (TC186449), two pyruvate decarboxylases (TC172484 and TC178207), and an acetyl-CoA synthetase (TC187142). The four upregulated transcripts belong to cluster 2, and two of them mapped to the introgression 10-1. Their overexpression could reflect an increased supply of acetyl-CoA, which suggests that carbohydrate pool depletion could contribute per se to lower ascorbate accumulation. The extra pool of acetyl-CoA could be channeled toward fatty acid biosynthesis or could increase the tricarboxylic acid flux. The first flux is supported by changes in the expression of transcripts annotated as stearoyl-acp desaturase (TC180054), fatty acid elongase-like protein (TC171885), nonspecific lipid transfer protein (TC172218), and wax synthase (TC177287 and TC182471). The second is supported by the upregulation of an NAD^+^-dependent isocitrate dehydrogenase subunit 1 (TC170372), of a glutamate decarboxylase (TC190777), of two tyrosine aminotransferases (TC180820 and TC190330), and an aspartate aminotransferase (TC190409), which comprehensively contribute to increased ROS production.

**Fig. 4:**
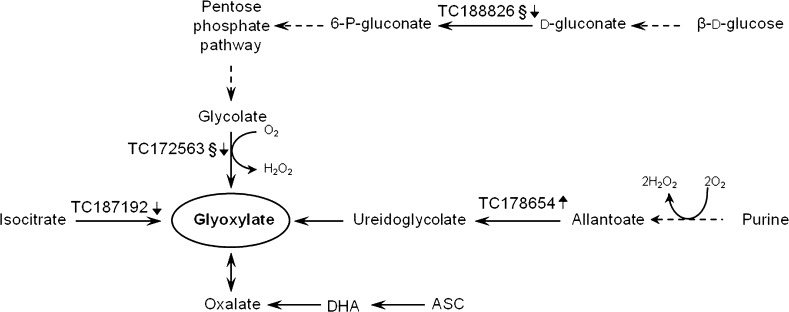
Network of genes mapping to glyoxylate metabolism, illustrating changes in their expression between IL10-1 and M82. §, Transcript mapping on the introgression 10-1. *DHA* dehydroascorbate, *ASC* ascorbate

Regarding glyoxylate metabolism (Fig. 4), the microarray experiment revealed the downregulation of a glycolate oxidase (TC172563) and a gluconokinase (TC188826), both mapping to the introgression 10-1 and probably involved in reducing the peroxisomal supply of glyoxylate. The key role of glycolate oxidase (TC172563) is also evidenced by its node position in the relevance network analysis. In addition, the downregulation of a sequence annotated as ATP-citrate lyase (TC187192) also suggests decreased synthesis of glyoxylate within the cytoplasm. On the other hand, our results point out the upregulation of an allantoate amidinohydrolase (TC178654), which is likely to increase the synthesis of glyoxylate from purine precursors, and this is also supported by the upregulation of a sequence annotated as endoribonuclease l-psp family protein (TC172641), mapping to the introgression and likely involved in supplying increasing amounts of purine intermediates to the peroxisomal ROS-producing catabolism. Thus, the reduced glyoxylate pool may be compensated through an increased breakdown of purines that operates through the hydrogen peroxide-producing xanthine oxidase. As a consequence, the increased synthesis of glyoxylate from purines rather than from glycolate would also increase the production of ROS by-products. Also, reduced synthesis of glyoxylate might be compensated via ascorbate catabolism from oxalate (Yu et al. 2010), which could further reduce the ascorbate pool. All these variations could lead to a lower ascorbate level if they occur in an NADH-limiting environment, as is the case in IL10-1 fruit where we observed the downregulation of NADH dehydrogenase (TC179606), suggesting a low NADH/NAD^+^ ratio in the cytoplasm. Furthermore, an increased ROS level in IL10-1 is supported by the upregulation of sequences involved in defense response (Supplementary Table 1).

Finally, a reduced ROS-scavenging activity is also suggested by the downregulation of two thioredoxins (TC177168 and TC185135, the latter mapping to the introgression) and three glutathione *S*-transferases (TC175968, TC189778, and TC182153). Therefore, changes in the expression of genes involved in increasing processes producing more ROS and downregulation of ROS-scavenging genes in an NADH-limited environment could lead to higher antioxidant demand that may contribute to lower ascorbate pool size. In addition, the depletion of carbohydrates, which are important ascorbate precursors, may also contribute to lowering the ascorbate pool in IL10-1 fruit.

## Discussion

As the most abundant antioxidant in plant tissues, ascorbate protects cells and organelles from oxidative damage by scavenging reactive oxygen species (Noctor and Foyer 1998) and is important for plant growth, stress resistance, maintenance of redox homeostasis, and as a signaling molecule involved in the regulation of plant response to environmental constraints (Pastori et al. 2003; Conklin and Barth 2004). Little is known about coordination and cross talk between genes and molecular mechanisms within ascorbate metabolism, their interaction with other biological processes, or how they control ascorbate biosynthesis and accumulation in tomato fruit. In a previous study (Di Matteo et al. 2010), we reported the fruit transcriptomic profiling of a tomato plant expressing a QTL for enhanced ascorbate content and highlighted a functional association between genes involved in cell wall catabolism, ethylene biosynthesis, and ascorbate pathways. Here, we report results from transcriptomic profiling of an additional IL (IL10-1) harboring a QTL with decreasing effect on fruit ascorbate content. Our results confirmed those previously reported by Rousseaux et al. (2005) and by Schauer et al. (2006).

Microarray analysis allowed us to identify a subset of genes differentially regulated in IL10-1 fruit and likely involved in reducing the ascorbate level. The model we proposed explains variation in ascorbate pool size in terms of changes in the steady-state level of specific mRNAs mainly occurring within glycolysis, fatty acid biosynthesis, glyoxylate metabolism, and the antioxidant system. Specifically, we propose that the reduced ascorbate concentration in the IL10-1 pericarp may result from depletion in the carbohydrate pool and increase in antioxidant demand. In particular, the upregulation of genes involved in glycolysis could result in decreasing the level of carbohydrate precursors thus limiting ascorbate biosynthesis. In fact, the influence of the carbohydrate pool size on ascorbate biosynthesis has long been established (Grace and Logan 1996). Also, the upregulation of genes involved in fatty acid biosynthesis and the tricarboxylic acid cycle in IL10-1 fruit suggested a competition between these pathways for acetyl-CoA and a decrease in the redox potential due to NADH reoxidization in fatty acid biosynthesis. Moreover, the detected upregulation of an NAD^+^-dependent isocitrate dehydrogenase subunit and other genes involved in the tricarboxylic acid cycle could reflect an increased tricarboxylic acid flux being generated from amino acid precursors. Indeed, this was in line with the higher soluble solid concentration in IL10-1 tomato fruit observed in our laboratory (data not shown) and already reported by Causse et al. (2004).

As for the antioxidant demand, our model proposes an increase in ROS levels in the IL10-1 fruit rising from a boost in ROS generation and a fall in the ROS-scavenging system. In particular, regulated processes leading to increased ROS generation in IL10-1 include (1) increased NADH level within the tricarboxylic acid cycle that might enhance electron leakage and ROS generation within the mitochondrion (Lenaz 2001; Hirst et al. 2008), (2) upregulated synthesis of glyoxylate from purines at the expense of the competing photorespiratory synthesis from glycolate in the peroxisome, and (3) reduced activity of antioxidant system components. According to our model, the decreased synthesis of glyoxylate via photorespiration and the glyoxylate cycle is partially compensated by purine via superoxide-producing xanthine oxidase where the synthesis occurs with a greater gain of ROS.

The increased antioxidant demand generated by ROS overproduction would imply a higher utilization of ascorbate and a possible effect on its overall level. Ascorbate can directly scavenge oxygen free radicals with and without enzyme catalysts (Shirahata et al. 1997; Halliwell and Gutteridge 2000) and can indirectly scavenge them by recycling tocopherol to its reduced form (Shao et al. 2008). Our results suggest that reduced strength of the ROS-scavenging system contributes to increased ROS levels and antioxidant demand in the IL10-1 fruit. Indeed, the reduced redox potential generated by the reoxidation of NADH in fatty acid biosynthesis could limit the efficiency of the Foyer–Halliwell–Asada cycle (Halliwell and Gutteridge 2000), which plays a key role in controlling ROS scavenging and the ascorbate level. Consistent with our hypothesis that the increase in ROS is the main determinant of ascorbate reduction in IL10-1 fruit, comparative profiling allowed us to identify a stress-related response presumably arising from peroxide accumulation, as also evidenced by the modified expression of many stress-related genes.

In conclusion, we propose that the ascorbate level in tomato fruit may be transcriptionally controlled by the expression of genes involved in carbohydrate catabolism, fatty acid biosynthesis, glyoxylate metabolism, and antioxidant system. Overall, the model proposed is intended to give a simplified meaning for molecular mechanisms controlling ascorbate accumulation in tomato fruit that have not been described so far. We report here for the first time that genes and processes operating outside the ascorbate biosynthetic pathways are effective in controlling the final concentration of ascorbate in a fruit system.

Consequently, candidate genes and molecular mechanisms identified may be relevant to biotechnological applications aimed at engineering the tomato for high fruit quality. Further attempts at enhancing ascorbate in tomato fruit could investigate the effectiveness of engineering glyoxylate metabolism, targeting, for instance, the cytoplasmic ATP-citrate lyase and the peroxisomal glycolate oxidase. Also, silencing genes controlling fatty acid synthesis or transmembrane transport, such as the lipid transport protein, may result in enhanced ascorbate levels in tomato fruit. By combining the overexpression of key genes in controlling the tricarboxylic acid cycle and key genes of glyoxylate metabolism, we expect to enhance soluble solid concentrations and ascorbate levels simultaneously.

## Electronic Supplementary Material

Below is the link to the electronic supplementary material.

Supplementary material 1 (DOC 306 kb)

Supplementary material 2 (PPT 182 kb)

Supplementary material 3 (PPT 106 kb)

Supplementary material 4 (PPT 103 kb)

Supplementary material 5 (TIFF 20240 kb)

Supplementary material 6 (PPT 260 kb)
