# Supplementary material for: Comparative Transcriptomic Profiling of Two Tomato Lines with Different Ascorbate Content in the Fruit
Source: Biochem Genet. 2012 Aug 22;50(11):908–21. doi: 10.1007/s10528-012-9531-3 (PMC3493670; doi:10.1007/s10528-012-9531-3)
Supplement: Supplementary file 1 — Supplementary material 1 (DOC 306 kb) [file 10528_2012_9531_MOESM1_ESM.doc]

**Supplementary Table 1.** Statistical and annotation details of 233 probes showing differential hybridization signals at a 2-factorial ANOVA (*P* < 0.01) in the IL10-1 transcriptome compared with M82. Statistical analysis was performed by the MultiExperiment Viewer *v*4.5 (MeV, part of the TM4 software); the IL 10-1 versus M82 fold change together with the adjusted *P* value were reported. Automatic Blast annotation of TC sequences was performed using Blast2GO software; the expectation (e) value, sequence similarity, and corresponding gene ontology terms were reported. TC transcripts listed in order of decreasing fold change between IL10-1 and M82. TC number followed by § indicates transcript physically mapping on introgression 10-1 of the tomato genome (release 2.40)

| Sequence ID | Fold change | P value | Annotation | Min. e value | Mean similarity (%) |
| --- | --- | --- | --- | --- | --- |
| TC172218§ | 5.46 | 5.61E-05 | nonspecific lipid transfer protein | 2.93E-52 | 89.10 |
| TC178207§ | 3.57 | 2.09E-04 | pyruvate decarboxylase | 0.00E+00 | 92.25 |
| TC190330§ | 3.24 | 1.14E-03 | tyrosine aminotransferase | 1.67E-78 | 80.25 |
| TC179186 | 3.20 | 1.86E-04 | pathogenesis-related protein | 3.56E-92 | 77.20 |
| TC180820§ | 3.15 | 4.69E-04 | tyrosine aminotransferase | 1.91E-102 | 83.45 |
| TC178746 | 2.82 | 3.39E-06 | protein tyrosine phosphatase | 9.70E-46 | 85.50 |
| TC172484§ | 2.44 | 9.74E-04 | pyruvate decarboxylase | 0.00E+00 | 89.15 |
| TC172641§ | 2.34 | 8.77E-05 | endoribonuclease l-psp family protein | 1.14E-98 | 82.15 |
| TC184006 | 2.30 | 4.84E-03 | cystathionine gamma-synthase | 1.04E-122 | 90.60 |
| TC178752 | 2.07 | 4.30E-03 | probable wound-induced protein | 1.96E-10 | 74.85 |
| TC181996 | 2.03 | 6.52E-03 | at1g67740 f12a21_13 | 2.30E-56 | 74.85 |
| TC190740 | 2.02 | 9.42E-03 | protein | 2.90E-166 | 74.95 |
| TC176881 | 1.87 | 8.67E-03 | glycine rich protein-interacting protein | 1.20E-32 | 75.00 |
| TC180054 | 1.82 | 2.65E-03 | stearoyl-acp desaturase | 1.36E-129 | 82.35 |
| TC186061 | 1.78 | 4.06E-03 | spx domain-containing protein | 3.24E-101 | 68.65 |
| TC190312 | 1.60 | 5.04E-03 | rna-dependent rna polymerase | 2.49E-153 | 96.75 |
| TC171116 | 1.58 | 8.92E-04 | ---NA--- |  |  |
| TC181425 | 1.48 | 6.15E-03 | 42kda chitin-binding protein | 6.87E-17 | 55.50 |
| TC189533 | 1.47 | 5.41E-03 | disease resistance identical | 4.76E-87 | 63.25 |
| TC171069 | 1.45 | 8.19E-03 | elicitor resposible protein | 1.85E-53 | 80.50 |
| TC170372§ | 1.43 | 3.54E-03 | nad+ dependent isocitrate dehydrogenase subunit 1 | 0.00E+00 | 91.70 |
| TC176245 | 1.42 | 1.91E-04 | protein | 2.50E-85 | 78.10 |
| TC170502§ | 1.39 | 9.54E-03 | acid phosphatase vanadium-dependent haloperoxidase-related protein | 1.94E-60 | 86.90 |
| TC182471 | 1.38 | 3.80E-03 | wax synthase | 9.90E-24 | 70.15 |
| TC170466 | 1.35 | 4.30E-03 | 33kda precursor protein of oxygen-evolving complex | 0.00E+00 | 93.90 |
| TC178008 | 1.35 | 5.20E-03 | plant synaptotagmin | 4.58E-87 | 89.75 |
| TC189569 | 1.33 | 6.14E-03 | predicted protein [*Populus trichocarpa*] | 3.55E-08 | 76.67 |
| TC177388 | 1.31 | 5.95E-03 | osmotin-like protein | 3.23E-153 | 94.30 |
| TC181500 | 1.30 | 1.41E-03 | universal stress protein family protein | 6.79E-66 | 84.60 |
| TC189087 | 1.29 | 6.57E-03 | amp dependent | 2.08E-109 | 86.75 |
| TC171432§ | 1.28 | 1.62E-03 | protein | 1.22E-157 | 91.60 |
| TC179899 | 1.25 | 2.52E-03 | protein kinase | 7.24E-45 | 63.85 |
| TC180762§ | 1.23 | 4.24E-03 | protein | 7.28E-86 | 86.50 |
| TC186401 | 1.23 | 6.71E-03 | alpha-tubulin | 3.19E-79 | 99.45 |
| TC177287 | 1.22 | 5.49E-03 | wax synthase | 1.08E-66 | 58.10 |
| TC184075 | 1.22 | 9.88E-03 | ---NA--- |  |  |
| TC181767 | 1.19 | 4.84E-03 | transducin wd-40 repeat-containing protein | 1.85E-81 | 86.75 |
| TC186647 | 1.17 | 8.30E-03 | ---NA--- |  |  |
| TC188293 | 1.15 | 3.82E-03 | serine threonine-protein phosphatase bsl3 | 4.19E-07 | 85.10 |
| TC184887 | 1.14 | 5.20E-03 | atp binding | 1.70E-44 | 69.25 |
| TC176268 | 1.12 | 8.77E-03 | protein | 8.64E-91 | 66.20 |
| TC191287 | 1.12 | 8.68E-03 | multidrug resistance protein abc transporter family | 6.28E-122 | 92.85 |
| TC182965 | 1.08 | 1.76E-03 | unnamed protein product [*Vitis vinifera*] | 2.40E-13 | 55.00 |
| TC191055 | 1.07 | 8.01E-03 | subtilisin-like protease | 1.12E-125 | 85.35 |
| TC186016 | 1.04 | 6.00E-03 | auxin efflux carrier-like protein | 5.00E-46 | 60.90 |
| TC174736 | 1.00 | 6.54E-03 | sulfate transporter | 1.14E-74 | 79.30 |
| TC185260§ | 0.99 | 2.55E-03 | transcription elongation factor 1 | 9.16E-33 | 91.35 |
| TC188023 | 0.98 | 7.94E-03 | flavonoid 3-hydroxilase | 0.00E+00 | 76.05 |
| TC190409 | 0.97 | 9.49E-04 | aspartate aminotransferase | 3.95E-39 | 96.00 |
| TC189209 | 0.95 | 8.38E-03 | cellulose synthase-like protein | 0.00E+00 | 90.70 |
| TC183957 | 0.92 | 3.95E-03 | uncharacterized protein | 2.94E-29 | 69.83 |
| TC173750 | 0.90 | 3.91E-04 | uncharacterized protein | 7.53E-19 | 50.33 |
| TC190465 | 0.90 | 4.22E-03 | squamosa-promoter binding protein | 8.46E-51 | 71.50 |
| TC190777 | 0.90 | 4.06E-03 | glutamate decarboxylase | 1.11E-96 | 89.05 |
| TC185275 | 0.89 | 2.56E-03 | hypothetical protein NitaMp040 [*Nicotiana tabacum*] | 4.42E-28 | 95.50 |
| TC179038§ | 0.89 | 2.98E-03 | thioredoxin m | 3.64E-41 | 76.15 |
| TC188296 | 0.87 | 3.66E-03 | protein | 9.54E-89 | 80.45 |
| TC180483 | 0.86 | 9.45E-03 | protein | 1.19E-58 | 70.45 |
| TC176520 | 0.86 | 9.28E-03 | host specificity protein j | 0.00E+00 | 99.00 |
| TC174512 | 0.85 | 2.10E-04 | ap2 erf domain-containing transcription factor | 5.78E-92 | 71.90 |
| TC179635 | 0.84 | 5.61E-03 | ---NA--- |  |  |
| TC185278 | 0.84 | 9.62E-03 | pollen-specific kinase partner protein | 4.48E-13 | 63.70 |
| TC181069 | 0.84 | 2.97E-03 | transcription factor tga1 | 8.01E-83 | 73.10 |
| TC186449 | 0.83 | 6.34E-03 | protein | 7.94E-131 | 90.40 |
| TC178654 | 0.77 | 8.55E-03 | allantoate amidohydrolase | 0.00E+00 | 81.10 |
| TC170189 | 0.76 | 4.16E-03 | protein | 4.08E-27 | 40.80 |
| TC176413 | 0.76 | 2.56E-03 | ankyrin repeat-containing | 1.39E-25 | 64.70 |
| TC173941 | 0.75 | 8.23E-03 | calcium ion binding | 0.00E+00 | 83.55 |
| TC171824 | 0.69 | 1.73E-03 | alanyl-trna synthetase | 1.46E-63 | 92.35 |
| TC170375 | 0.69 | 6.27E-03 | vacuolar cation proton exchanger 1 | 1.82E-95 | 82.00 |
| TC187142 | 0.68 | 5.38E-03 | protein | 0.00E+00 | 74.95 |
| TC172065 | 0.61 | 2.43E-03 | protein | 8.92E-88 | 78.50 |
| TC186583 | 0.57 | 2.43E-03 | uncharacterized protein | 2.37E-13 | 90.67 |
| TC174691 | 0.57 | 4.45E-03 | peroxidase atp19a | 2.21E-24 | 70.15 |
| TC179425 | 0.55 | 6.87E-03 | amine oxidase | 9.00E-61 | 75.90 |
| TC171101 | 0.50 | 7.51E-03 | protein | 1.35E-71 | 72.85 |
| TC176435 | 0.47 | 6.62E-04 | amino acid permease | 3.06E-74 | 75.85 |
| TC176921 | 0.45 | 8.83E-03 | serine-threonine protein plant- | 2.35E-115 | 70.85 |
| TC187242 | 0.44 | 8.24E-03 | cytoplasmic trna 2-thiolation protein 1 | 1.18E-121 | 92.40 |
| TC188913 | 0.39 | 8.96E-03 | at1g52190-like protein | 5.12E-54 | 71.60 |
| TC171304 | 0.39 | 1.70E-03 | protein | 7.97E-96 | 89.90 |
| TC185606 | 0.38 | 1.58E-03 | regulator of chromosome condensation repeat-containing protein | 5.00E-68 | 80.94 |
| TC176013 | 0.35 | 8.29E-03 | serine-threonine protein plant- | 8.61E-69 | 66.60 |
| TC190088 | 0.29 | 1.64E-03 | rna recognition motif-containing protein | 1.98E-46 | 67.25 |
| TC174699 | 0.04 | 6.90E-03 | PREDICTED: hypothetical protein [*Vitis vinifera*] | 1.82E-05 | 56.17 |
| TC175154 | -0.01 | 8.22E-03 | protein | 5.70E-85 | 78.25 |
| TC181489 | -0.09 | 3.26E-03 | 5-amp-activated protein kinase-like protein | 4.47E-21 | 63.40 |
| TC178778§ | -0.25 | 9.07E-03 | uncharacterized protein | 3.49E-47 | 78.10 |
| TC182760 | -0.27 | 1.11E-03 | mitochondrial processing peptidase | 0.00E+00 | 88.15 |
| TC190804 | -0.28 | 6.97E-03 | photosystem ii 47 kda protein | 0.00E+00 | 99.70 |
| TC183091 | -0.33 | 3.67E-03 | uncharacterized protein | 3.50E-140 | 78.15 |
| TC186798 | -0.42 | 4.31E-03 | at4g33690 t16l1_180 | 1.54E-58 | 65.40 |
| TC176732 | -0.43 | 4.11E-03 | uncharacterized protein | 6.91E-120 | 74.85 |
| TC182527 | -0.44 | 2.30E-03 | like cov 2 protein | 6.55E-72 | 87.95 |
| TC177519 | -0.46 | 5.33E-03 | atp binding | 1.02E-41 | 71.40 |
| TC171139 | -0.47 | 7.19E-03 | s-adenosylmethionine-dependent methyltransferase domain-containing protein | 1.50E-127 | 82.75 |
| TC188537 | -0.47 | 8.54E-03 | protein abil1 | 1.05E-17 | 76.00 |
| TC179473 | -0.48 | 9.73E-03 | clathrin adapter complex medium subunit-like protein | 8.06E-66 | 83.70 |
| TC175863 | -0.49 | 2.65E-03 | enolase | 0.00E+00 | 94.95 |
| TC179043 | -0.50 | 7.19E-03 | rna recognition motif family expressed | 1.06E-46 | 74.75 |
| TC177617§ | -0.50 | 3.27E-03 | adenine nucleotide alpha hydrolases-like protein | 1.45E-68 | 79.65 |
| TC170194 | -0.51 | 2.28E-03 | protein tyrosine expressed | 0.00E+00 | 78.10 |
| TC172285 | -0.52 | 7.11E-03 | beta chain | 0.00E+00 | 98.80 |
| TC183614 | -0.54 | 6.75E-03 | protein | 1.26E-86 | 84.20 |
| TC172391 | -0.54 | 7.89E-03 | uncharacterized protein | 7.74E-101 | 85.35 |
| TC181864 | -0.57 | 5.09E-03 | phosphatidic acid phosphatase family protein | 7.88E-113 | 77.85 |
| TC180796 | -0.58 | 1.76E-03 | phospholipase c | 1.53E-163 | 79.35 |
| TC182662 | -0.59 | 2.70E-03 | protein | 6.70E-116 | 75.95 |
| TC179606§ | -0.60 | 4.39E-03 | nadh dehydrogenase | 7.14E-147 | 85.25 |
| TC170714§ | -0.63 | 1.73E-03 | gamma-glutamyl hydrolase 2 | 1.41E-151 | 81.65 |
| TC173198 | -0.65 | 9.75E-03 | serine threonine protein kinase | 7.79E-152 | 82.20 |
| TC173720 | -0.67 | 4.75E-03 | 30s ribosomal protein s13 | 6.53E-58 | 85.00 |
| TC179455 | -0.67 | 4.59E-03 | o-fucosyltransferase family protein | 4.21E-158 | 75.55 |
| TC175010 | -0.68 | 9.77E-03 | protein | 2.58E-112 | 77.60 |
| TC184002 | -0.70 | 7.37E-03 | endonuclease 3 | 7.47E-48 | 73.40 |
| TC186383 | -0.72 | 8.73E-03 | heptahelical transmembrane protein2 | 3.60E-64 | 76.65 |
| TC181889 | -0.72 | 1.57E-03 | protein | 6.05E-53 | 75.25 |
| TC182124 | -0.73 | 5.02E-03 | aminopeptidase c | 1.30E-167 | 76.95 |
| TC176636§ | -0.74 | 8.46E-03 | cmp-2-keto-3-deoctulosonate (cmp-kdo) | 3.88E-115 | 91.75 |
| TC189515 | -0.75 | 7.38E-03 | histone acetyltransferase | 1.93E-21 | 87.60 |
| TC184105 | -0.76 | 9.48E-03 | ngg1 interacting factor 3 like 1 binding protein 1 isoform 1 | 2.14E-99 | 90.05 |
| TC178789§ | -0.78 | 9.37E-04 | protein | 5.13E-68 | 64.45 |
| TC177707 | -0.78 | 3.31E-03 | 40s ribosomal protein s28 | 3.36E-19 | 94.25 |
| TC181284 | -0.79 | 7.74E-03 | protein | 4.26E-34 | 76.30 |
| TC187906 | -0.79 | 2.14E-03 | nonimprinted in prader-willi angelman syndrome region | 8.04E-17 | 78.58 |
| TC186025 | -0.81 | 6.29E-03 | uncharacterized protein | 1.05E-33 | 81.00 |
| TC187192 | -0.82 | 4.96E-03 | atp-citrate lyase a-3 | 0.00E+00 | 94.00 |
| TC172857 | -0.83 | 6.60E-03 | sigma factor sigb regulation protein | 1.16E-128 | 89.35 |
| TC174331 | -0.83 | 8.74E-03 | pollen-specific c2 domain containing protein | 8.87E-88 | 90.25 |
| TC184557 | -0.85 | 3.28E-04 | phd finger protein | 2.02E-71 | 80.40 |
| TC190377 | -0.88 | 2.01E-03 | rab family gtpase | 8.49E-12 | 92.25 |
| TC179412 | -0.88 | 4.02E-03 | cationic amino acid transporter | 2.56E-66 | 83.25 |
| TC173762§ | -0.90 | 4.78E-03 | gamma-glutamyl hydrolase 2 | 1.57E-66 | 79.75 |
| TC180189 | -0.91 | 5.72E-05 | protein | 9.27E-26 | 87.80 |
| TC174842§ | -0.94 | 3.61E-03 | uncharacterized protein | 2.43E-128 | 85.80 |
| TC182861 | -0.94 | 4.52E-03 | aldehyde dehydrogenase | 7.52E-54 | 92.00 |
| TC189143 | -0.94 | 8.94E-04 | protein phosphatase 2c | 3.21E-54 | 74.75 |
| TC182858 | -0.97 | 9.35E-03 | vacuolar-sorting receptor 7 | 7.15E-164 | 83.50 |
| TC179122 | -1.00 | 5.78E-03 | uncharacterized protein | 2.17E-100 | 72.50 |
| TC177895 | -1.00 | 9.78E-03 | uncharacterized protein | 4.82E-97 | 83.05 |
| TC190844 | -1.02 | 6.21E-03 | cytochrome p450 | 1.24E-136 | 72.30 |
| TC182917 | -1.04 | 1.08E-04 | inner membrane protein albino3 | 5.82E-179 | 82.75 |
| TC185662 | -1.04 | 1.79E-03 | phd finger protein | 5.85E-29 | 92.80 |
| TC176914 | -1.07 | 4.80E-03 | 60s ribosomal protein l30 | 2.79E-81 | 84.60 |
| TC177536 | -1.08 | 5.08E-04 | protein | 2.53E-84 | 80.10 |
| TC176470 | -1.08 | 7.07E-03 | ribosomal protein s21 family protein | 1.28E-33 | 79.61 |
| TC170674 | -1.09 | 3.05E-03 | expansin precursor | 1.69E-140 | 83.15 |
| TC171885 | -1.11 | 9.76E-03 | hxxxd-type acyl-transferase-like protein | 2.55E-124 | 62.00 |
| TC178286§ | -1.13 | 6.91E-03 | btb poz domain-containing protein | 5.16E-138 | 90.95 |
| TC189970§ | -1.16 | 1.70E-03 | atp binding | 6.51E-151 | 83.30 |
| TC186674 | -1.18 | 8.56E-03 | uncharacterized protein | 7.46E-50 | 52.95 |
| TC185528§ | -1.18 | 2.57E-03 | uncharacterized protein | 2.79E-53 | 71.21 |
| TC176227§ | -1.19 | 7.97E-03 | rna recognition motif-containing protein | 9.87E-22 | 65.23 |
| TC179931 | -1.19 | 7.71E-03 | ---NA--- |  |  |
| TC179755 | -1.20 | 5.66E-03 | calcium ion binding | 3.59E-97 | 59.45 |
| TC186472§ | -1.24 | 3.86E-03 | protein bps1 | 2.18E-123 | 78.10 |
| TC175617§ | -1.24 | 3.01E-03 | calmodulin-domain protein kinase cdpk isoform 5 | 4.33E-128 | 95.65 |
| TC182944 | -1.24 | 2.22E-05 | tetratricopeptide repeat domain-containing protein | 8.80E-57 | 72.45 |
| TC174974§ | -1.24 | 1.55E-03 | cdp-diacylglycerol--glycerol-3-phosphate 3- | 6.53E-48 | 75.56 |
| TC177552§ | -1.27 | 6.06E-05 | nudix hydrolase 15 | 3.06E-77 | 70.60 |
| TC188592§ | -1.28 | 2.94E-03 | ---NA--- |  |  |
| TC171109§ | -1.29 | 6.34E-04 | 30s ribosomal protein s10 | 9.48E-63 | 80.70 |
| TC180327§ | -1.29 | 2.08E-03 | adenosine deaminase | 1.13E-120 | 74.20 |
| TC188826§ | -1.30 | 1.61E-03 | gluconokinase | 5.47E-59 | 73.40 |
| TC183973 | -1.31 | 7.68E-03 | ---NA--- |  |  |
| TC183000§ | -1.38 | 4.47E-04 | thylakoid membrane phosphoprotein | 3.43E-45 | 65.30 |
| TC175968 | -1.39 | 4.19E-03 | glutathione s-transferase t3 | 8.72E-128 | 73.35 |
| TC189749 | -1.40 | 9.72E-03 | cytochrome p450 | 1.19E-77 | 88.40 |
| TC175531 | -1.41 | 4.51E-03 | basic 7s globulin 2 precursor small | 3.39E-36 | 75.55 |
| TC174717 | -1.42 | 1.31E-03 | beta chain | 2.81E-87 | 99.30 |
| TC179505 | -1.45 | 6.00E-06 | endomembrane protein emp70 precusor isolog | 4.98E-125 | 96.20 |
| TC172875 | -1.47 | 9.34E-03 | ap2 domain cbf protein | 9.53E-99 | 86.20 |
| TC173618 | -1.56 | 2.84E-03 | protein | 1.48E-33 | 70.90 |
| TC183263§ | -1.63 | 1.25E-03 | rna recognition motif-containing protein | 5.49E-142 | 77.40 |
| TC186405§ | -1.64 | 7.92E-04 | protein | 1.56E-126 | 75.20 |
| TC181144 | -1.65 | 2.99E-04 | pectinacetylesterase family protein | 3.44E-99 | 84.30 |
| TC189885 | -1.67 | 7.23E-05 | RRP6-like protein | 1.12E-23 | 49.80 |
| TC175950§ | -1.67 | 7.05E-03 | rna recognition motif -containing protein | 1.75E-105 | 80.70 |
| TC178443 | -1.68 | 4.36E-03 | PREDICTED: hypothetical protein [*Vitis vinifera*] | 1.28E-06 | 78.67 |
| TC186848 | -1.75 | 3.99E-03 | transcription factor iiia | 2.97E-139 | 75.15 |
| TC177320§ | -1.76 | 2.80E-03 | vacuolar sorting receptor 1 | 4.67E-132 | 93.50 |
| TC179572§ | -1.79 | 1.17E-04 | protein | 1.72E-44 | 62.85 |
| TC187253 | -1.81 | 4.32E-04 | asr4 protein | 2.15E-98 | 68.50 |
| TC170015 | -1.81 | 1.90E-04 | rubber elongation factor | 1.30E-99 | 72.85 |
| TC180320§ | -1.82 | 1.47E-04 | apoptosis inhibitory protein 5 | 2.85E-91 | 76.30 |
| TC181386 | -1.87 | 4.10E-03 | uncharacterized protein | 1.21E-34 | 57.80 |
| TC171817 | -1.91 | 1.16E-03 | predicted protein [*Populus trichocarpa*] | 7.44E-51 | 59.45 |
| TC183917 | -1.92 | 6.50E-03 | exostosin-like protein | 2.15E-08 | 74.38 |
| TC174925§ | -1.93 | 7.03E-05 | rna recognition motif-containing protein | 5.76E-62 | 80.25 |
| TC175785 | -1.98 | 7.15E-03 | ap2 domain cbf protein | 6.70E-109 | 87.05 |
| TC187509 | -1.99 | 7.97E-04 | ubiquitin-conjugating enzyme | 6.57E-94 | 91.90 |
| TC174656 | -2.03 | 9.14E-03 | uncharacterized protein | 2.90E-27 | 83.65 |
| TC183910 | -2.03 | 9.28E-03 | protein | 0.00E+00 | 84.75 |
| TC177168 | -2.05 | 6.04E-06 | thioredoxin family protein | 1.20E-99 | 77.75 |
| TC181792§ | -2.06 | 3.69E-04 | yippee-like protein | 6.34E-47 | 81.50 |
| TC186231 | -2.10 | 5.33E-03 | magnesium-protoporphyrin ix monomethyl ester | 0.00E+00 | 91.00 |
| TC186308§ | -2.10 | 1.35E-03 | vacuolar sorting receptor protein | 4.16E-14 | 83.70 |
| TC172996§ | -2.17 | 5.22E-03 | histone h2a | 1.11E-52 | 94.95 |
| TC175513 | -2.22 | 2.09E-03 | v-type h+-transporting atpase 16kda proteolipid subunit | 9.65E-56 | 98.20 |
| TC186907 | -2.22 | 3.08E-03 | f-box family protein | 6.54E-91 | 76.70 |
| TC173311 | -2.24 | 4.10E-04 | gns1 sur4 membrane family protein | 1.71E-102 | 72.25 |
| TC175109§ | -2.25 | 8.89E-04 | prohibitin-like protein | 2.71E-154 | 94.65 |
| TC173447§ | -2.26 | 1.55E-05 | predicted protein [*Nicotiana tabacum*] | 1.66E-05 | 44.00 |
| TC181272§ | -2.35 | 2.91E-04 | ob-fold nucleic acid binding domain containing protein | 1.63E-46 | 78.20 |
| TC182647§ | -2.35 | 5.31E-05 | protein | 1.63E-84 | 88.85 |
| TC185607§ | -2.37 | 5.71E-05 | glutaryl-dehydrogenase | 1.28E-133 | 90.40 |
| TC171426§ | -2.42 | 1.45E-05 | isoamyl acetate-hydrolyzing | 3.93E-84 | 75.85 |
| TC184093§ | -2.43 | 6.53E-05 | dna-directed rna polymerases and iii kda polypeptide | 1.79E-21 | 92.65 |
| TC179403 | -2.46 | 1.30E-04 | transcriptional coactivator-like protein | 4.38E-59 | 95.95 |
| TC176198§ | -2.66 | 5.03E-05 | protein | 1.12E-82 | 98.10 |
| TC172352§ | -2.74 | 2.15E-04 | aterf3 erf3 | 1.32E-34 | 76.00 |
| TC178916 | -2.80 | 2.04E-05 | glycine-rich rna-binding protein | 6.29E-36 | 92.85 |
| TC172404§ | -2.83 | 2.84E-04 | ribose-phosphate pyrophosphokinase 4 | 2.30E-158 | 88.25 |
| TC176093 | -2.84 | 6.35E-04 | peroxisomal biogenesis factor 11 family protein | 4.65E-118 | 90.25 |
| TC170557 | -2.93 | 1.78E-03 | phenylcoumaran benzylic ether reductase 3 | 1.70E-137 | 79.10 |
| TC176896 | -3.05 | 3.78E-05 | mip sip subfamily | 6.83E-56 | 87.30 |
| TC176706§ | -3.17 | 7.29E-08 | quercetin 3-o-glucoside-6 -o-malonyltransferase | 1.27E-45 | 59.80 |
| TC185135§ | -3.40 | 4.65E-05 | thioredoxin m | 9.21E-45 | 84.00 |
| TC184292§ | -3.49 | 1.37E-06 | transducin wd40 domain-containing protein | 0.00E+00 | 87.45 |
| TC181468 | -3.75 | 6.18E-05 | inorganic pyrophosphatase | 6.66E-133 | 84.70 |
| TC185161 | -3.78 | 7.22E-03 | protein | 2.50E-31 | 65.65 |
| TC176740§ | -3.88 | 2.85E-07 | uncharacterized protein | 1.24E-101 | 78.40 |
| TC176172 | -3.91 | 4.49E-03 | protein | 1.80E-54 | 56.65 |
| TC173444§ | -4.21 | 9.09E-03 | adenosine deaminase | 7.44E-61 | 75.25 |
| TC170197 | -4.65 | 1.75E-05 | tim17 domain-containing protein | 9.71E-57 | 80.95 |
| TC189470 | -4.94 | 2.29E-05 | nitrite reductase | 7.88E-142 | 93.55 |
| TC173934 | -5.64 | 6.99E-05 | cytochrome p450 monooxygenase | 0.00E+00 | 79.85 |
| TC174357§ | -6.04 | 6.49E-03 | uroporphyrinogen decarboxylase 1 | 1.37E-174 | 84.35 |
| TC189778 | -6.40 | 6.42E-03 | glutathione s-transferase gst 14 | 5.14E-29 | 77.00 |
| TC172563§ | -6.85 | 3.71E-05 | glycolate oxidase | 3.56E-180 | 93.65 |
| TC173893§ | -8.27 | 2.06E-06 | ---NA--- |  |  |
| TC185597 | -8.70 | 2.32E-05 | ---NA--- |  |  |
| TC182153 | -9.76 | 1.43E-03 | glutathione s-transferase | 1.02E-49 | 82.00 |

**SUPPLEMENTARY FIGURE LEGEND**

Supplementary Fig. 4. Hierarchical clustering and the correlation among differentially expressed signals in M82 and IL10-1 fruit transcriptomes (based on Pearson correlation). Color scale (top) ranges from 0.0 (green) to 682.81067 (black) to 1868.4265 (red). Clusters are indicated on the left of the column. Data for two years are shown, with 2007 on the left and 2008 on the right, and M82 is shown first under each year. TC transcripts are identified by number and function on the right of the column. Cluster 2 contains upregulated sequences in IL10-1 fruit linking genes involved in carbohydrate (glycolysis and the citric acid cycle) and amino acid catabolism with ethylene and defense responses. Cluster 5 mainly includes sequences related to peroxisome metabolism and biogenesis, such as a glycolate oxidase and a peroxisomal biogenesis factor
