# Supplementary material for: Comparative Transcriptomic Profiling of Two Tomato Lines with Different Ascorbate Content in the Fruit
Source: Biochem Genet. 2012 Aug 22;50(11):908–21. doi: 10.1007/s10528-012-9531-3 (PMC3493670; doi:10.1007/s10528-012-9531-3)
Supplement: Supplementary file 2 — Supplementary material 2 (PPT 182 kb) [file 10528_2012_9531_MOESM2_ESM.ppt]

## Slide 1
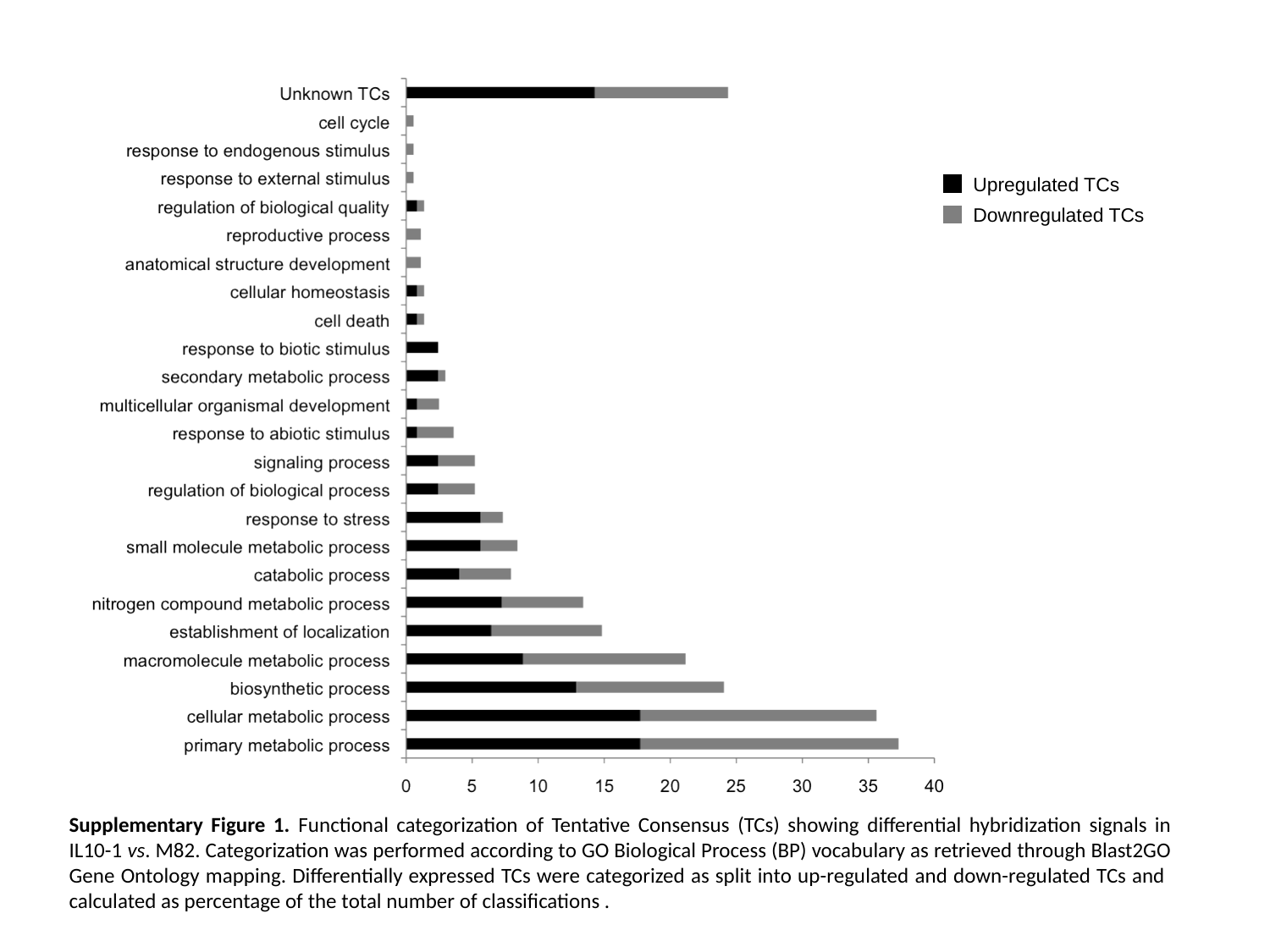

Upregulated TCs
Downregulated TCs
Supplementary Figure 1. Functional categorization of Tentative Consensus (TCs) showing differential hybridization signals in IL10-1 vs. M82. Categorization was performed according to GO Biological Process (BP) vocabulary as retrieved through Blast2GO Gene Ontology mapping. Differentially expressed TCs were categorized as split into up-regulated and down-regulated TCs and calculated as percentage of the total number of classifications .
