# Supplementary figures and images for: Comparative Transcriptomic Profiling of Two Tomato Lines with Different Ascorbate Content in the Fruit
Source: Biochem Genet. 2012 Aug 22;50(11):908–21. doi: 10.1007/s10528-012-9531-3 (PMC3493670; doi:10.1007/s10528-012-9531-3)

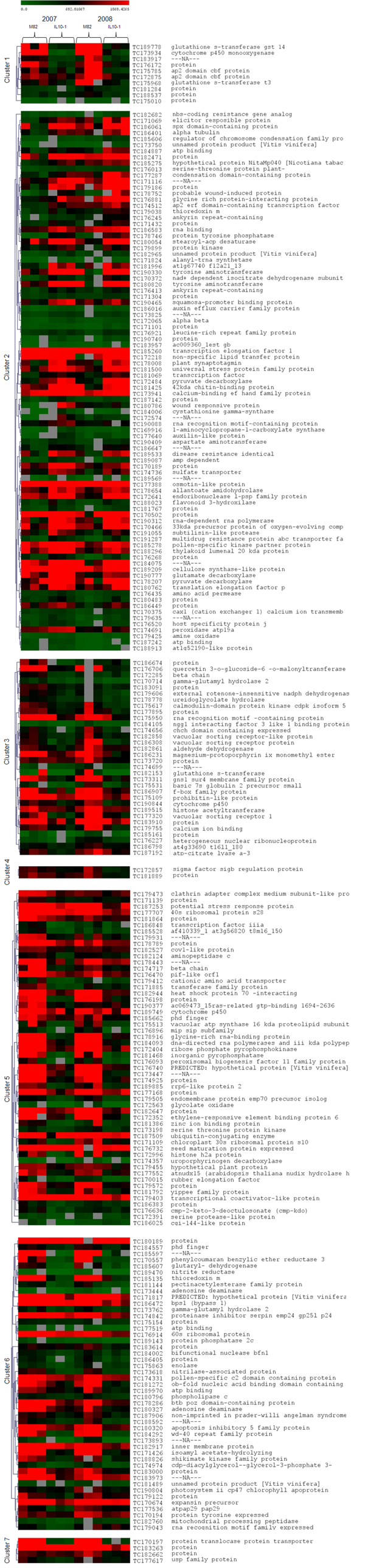

Supplement: Supplementary file 5 — Supplementary material 5 (TIFF 20240 kb) [file 10528_2012_9531_MOESM5_ESM.tiff]
