# Supplementary material for: Comparative Transcriptomic Profiling of Two Tomato Lines with Different Ascorbate Content in the Fruit
Source: Biochem Genet. 2012 Aug 22;50(11):908–21. doi: 10.1007/s10528-012-9531-3 (PMC3493670; doi:10.1007/s10528-012-9531-3)
Supplement: Supplementary file 6 — Supplementary material 6 (PPT 260 kb) [file 10528_2012_9531_MOESM6_ESM.ppt]

## Slide 1
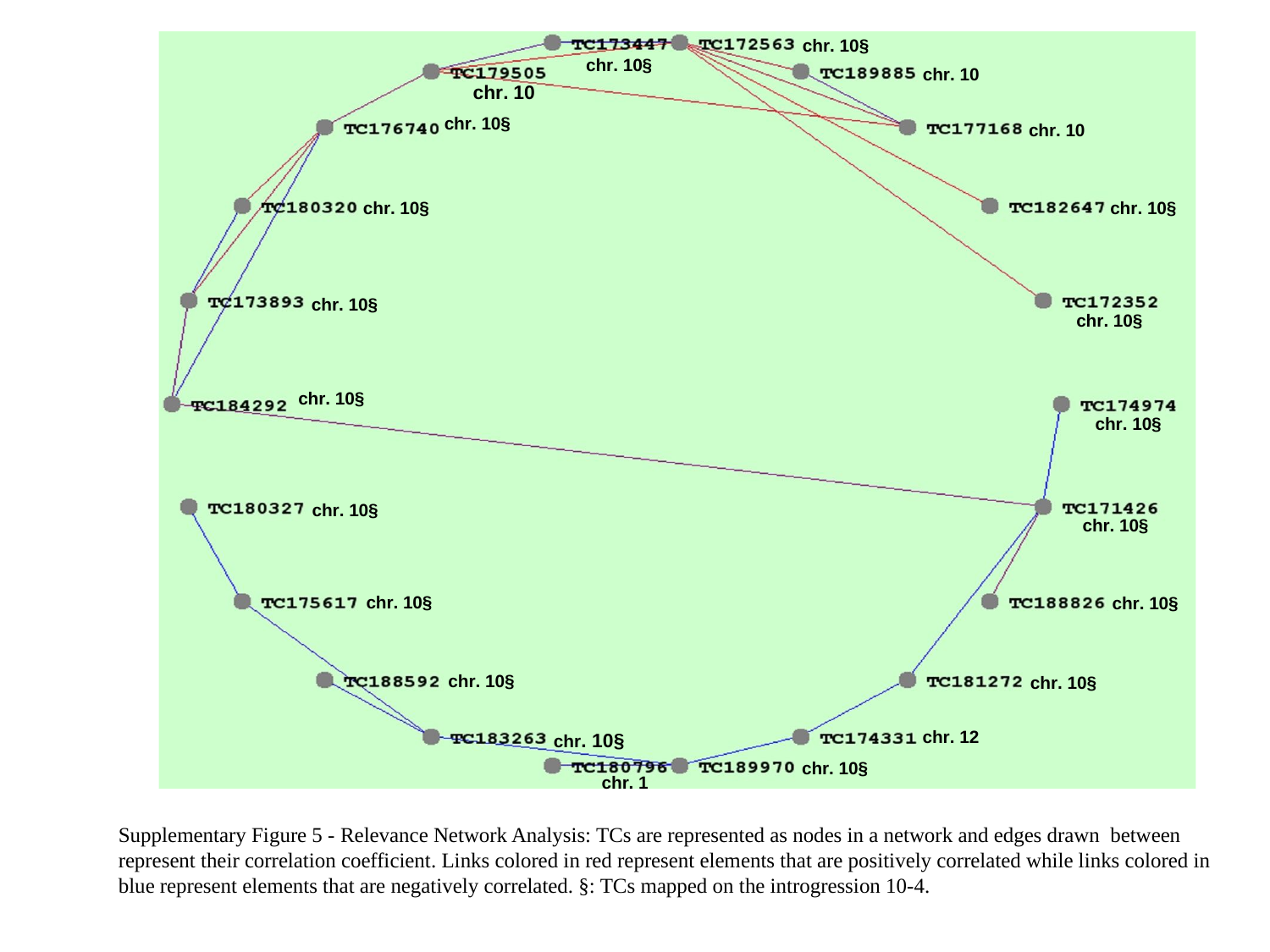

chr. 10§
chr. 10§
chr. 10
chr. 10
chr. 10§
chr. 10
chr. 10§
chr. 10§
chr. 10§
chr. 10§
chr. 10§
chr. 10§
chr. 10§
chr. 10§
chr. 10§
chr. 10§
chr. 10§
chr. 10§
chr. 12
chr. 10§
chr. 10§
chr. 1
Supplementary Figure 5 - Relevance Network Analysis: TCs are represented as nodes in a network and edges drawn between represent their correlation coefficient. Links colored in red represent elements that are positively correlated while links colored in blue represent elements that are negatively correlated. §: TCs mapped on the introgression 10-4.
